# Substance use and the value of control

**DOI:** 10.3758/s13415-026-01451-z

**Published:** 2026-05-06

**Authors:** Elle M. Giovanni, Mimi Liljeholm

**Affiliations:** 1 Department of Cognitive Sciences, University of California, Irvine, CA USA; 2 Irvine Center for Addiction Neuroscience, University of California, Irvine, CA USA; 3 Center for the Neurobiology of Learning & Memory, University of California, Irvine, CA USA

**Keywords:** Substance use, Abstinence, Intrinsic motivation, Instrumental control, Subjective utility

## Abstract

Clinical criteria for substance-use disorder collectively specify a loss of instrumental control over consumption; yet little empirical work has addressed how substance use shapes the utility of agency in motivated behavior. We combined a hierarchical gambling task with cross-sectional substance-use surveys and computational cognitive modeling to assess the preference for controllable environments in adult humans with self-reported abstinence from psychostimulants, opioids, alcohol, or sedatives. For psychostimulant use, longer abstinence selectively increased the preference for instrumental control. In contrast, for opioids and alcohol, longer abstinence respectively predicted increased preferences for divergent outcome distributions and free choice, regardless of controllability. These associations between substance use and decision making were dissociable from participants’ ability to maximize monetary payoffs in controllable environments. Our findings implicate the dopaminergic system in agency coding.

**Supplementary Information:**

The online version contains supplementary material available at https://doi.org/10.3758/s13415-026-01451-z.

## Introduction

Despite a longstanding debate regarding the legal and ethical implications of characterizing addiction as a voluntary choice (Brown, 2019), very little is known about how psychoactive substance use modulates the representation and utilization of agency. Neurotypical human adults (Bobadilla-Suarez et al., 2017; Bown et al., 2003; Leotti & Delgado, 2011; Leotti et al., 2010; Suzuki, 1997) and a range of nonhuman animals (Catania, 1975; Perdue et al., 2014; Shuji Suzuki, 1999; Voss & Homzie, 1970) have a well-demonstrated preference for free over forced choice. This preference may confer selective advantage: Because the subjective utilities of sensory stimuli (e.g., a particular food or piece of music) fluctuate continuously according to hedonic and homeostatic dynamics, reward maximization requires an ability to flexibly produce distinct sensory states according to their current utilities (Liljeholm, 2018, 2021, 2022). To better characterize the role of agency dysregulation in addiction, we assessed whether individual differences in the duration of self-reported abstinence from substance use predicted the subjective utility of instrumental control across a range of psychoactive substances.

**Fig. 1:**
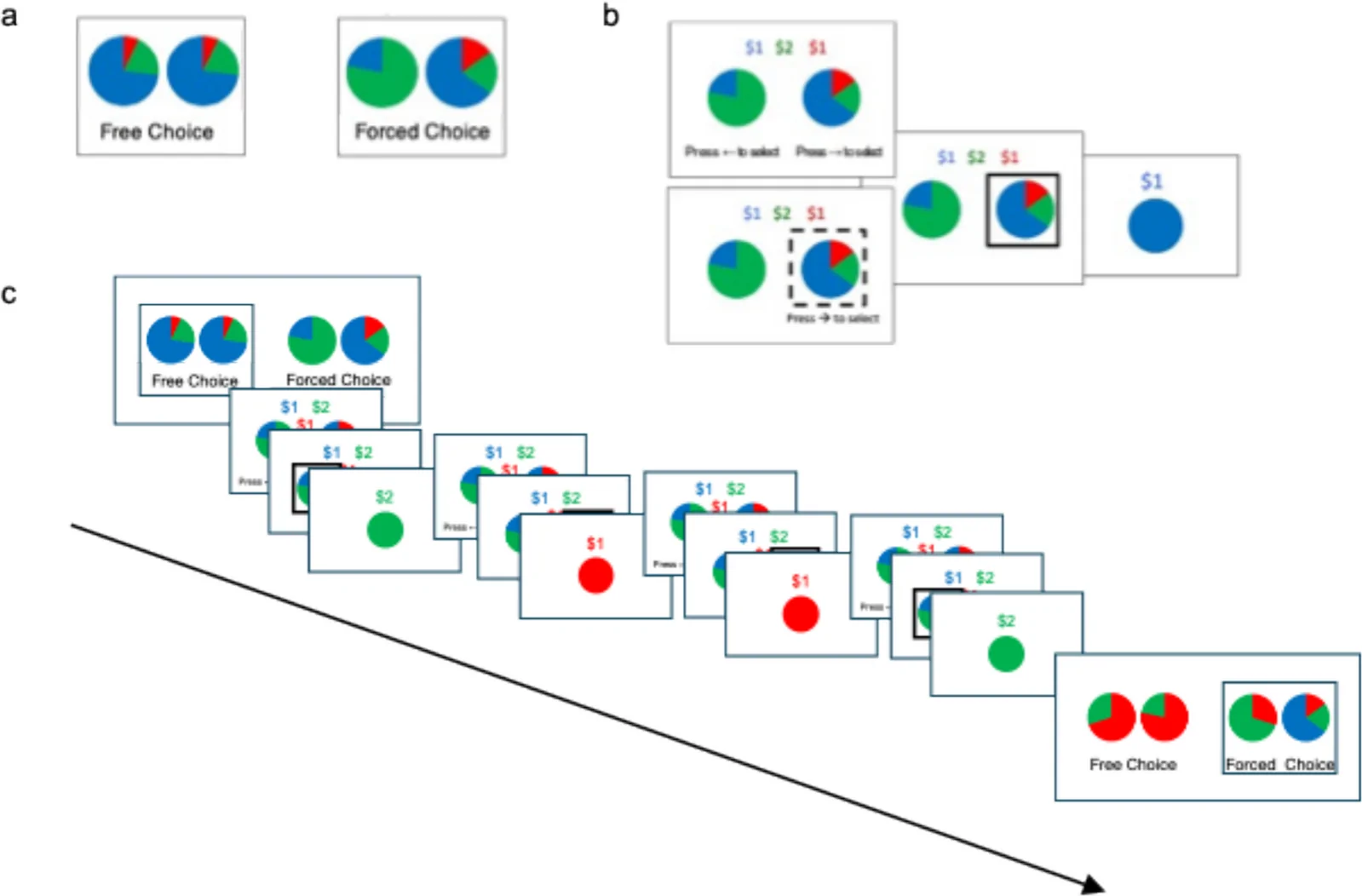
Task illustration. *Note.* (**a**) Two gambling ‘rooms’ with low (Left) and high (Right) outcome divergence, neither of which affords instrumental control. (**b**) Choice, selection, and feedback screens on a trial inside a gambling room, illustrating both free (top) and forced (bottom) choice screens. (**c**) Task-flow excerpt, showing a room choice, followed by four gambling trials in the selected room, and the subsequent room choice. There were 48 room choices, each followed by four gambling trials

As an illustration of an environment’s controllability, consider the scenario depicted in Fig. 1a, where each of two “gambling rooms” contains two gambling options, and each option has a probability distribution over three outcome colors, as indicated by the pie charts. Assume that each color will be subsequently associated with an arbitrary amount of monetary loss or gain and that you must select a room to gamble in *before* the monetary color values are revealed, mimicking the volatility of subjective sensory utilities. Which room would you prefer?

In the left room, although afforded free choice, you would have very little control over which color, and thus which monetary outcome, was produced by your selections, because color outcome distributions are identical across options. Conversely, in the right room, although color outcome distributions diverge across gambling options, you are forced to accept the selections of a computer algorithm that alternates between options across trials, again eliminating instrumental control. However, had you been afforded Free Choice in the room on the right side of Fig. 1a, its divergent outcome distributions would have allowed you to completely avoid the red outcome or to dramatically switch between green and blue outcomes, according to subsequently revealed monetary color values.

Consistent with this constraint, neurotypical adults prefer environments in which freely chosen options produce divergent outcome distributions—i.e., those that are *controllable* (Liljeholm, 2022; Liljeholm et al., 2018; Mistry & Liljeholm, 2016; Norton & Liljeholm, 2020)—over those with free choice or outcome divergence alone. Intriguingly, such preferences are negatively correlated with the degree of positive schizotypy (Liljeholm, 2022; Liljeholm et al., 2018)—a subclinical analogue of positive schizophrenia, characterized by unusual thoughts and beliefs (Mason & Claridge, 2006). As with schizophrenia, positive schizotypy is heavily associated with cortico-striatal dopamine (DA) function (Dawe et al., 2013; Mohr & Ettinger, 2014; Woodward et al., 2011), and reliably predicts reductions in subjective experiences of agency, whether explicitly or implicitly assessed (Asai, 2016; Rossetti et al., 2024; Szendi et al., 2024). Together, these findings suggest that illicit use of indirect dopamine agonists, most notably cocaine and amphetamine, may dysregulate representational and/or motivational aspects of human agency. Here, we assessed whether the preference for controllable environments is modulated by the recency of psychostimulant, opioid, alcohol, and/or sedative use.

## Methods

### Participants

We identified and invited 4,059 eligible volunteers to participate for monetary compensation via ResearchMatch.org, an NIH-funded clinical and nonclinical recruitment platform where subjects can self-identify with various conditions and life experiences. We used the ResearchMatch search function to query the database with substance- (e.g., amphetamine, alcohol, heroin) and addiction-related (e.g., addiction, substance abuse) keywords to identify participants with active or past use of psychostimulants, opioids, alcohol, and/or sedatives, allowing for polysubstance use. Participants’ self-reported history of use of the four screened substances served as the criterion for study inclusion (i.e., for subsequent contact by the experimenter). Of 382 that accepted the invitation and were sent the study link, 133 (75 females; mean age = 38.4 ± 13 years) completed the study in a single online session for a baseline payment of $7. In addition to the base payment, participants could receive up to $15 according to experimental contingencies. All participants gave informed consent and the study conformed with the ethical principles outlined in the Declaration of Helsinki. The institutional review board of the University of California, Irvine approved the study.

### Substance use assessment

Four categories of substances were screened: psychostimulants (e.g., cocaine/crack, amphetamine), opioids (e.g., heroin, fentanyl, hydrocodone), sedatives (e.g., barbiturates, benzodiazepines), and alcohol. Our primary predictor was abstinence from substance, as this is a well-established predictor of substance-induced dysregulation of reward-based decision making (Bolla et al., 2005; Körner et al., 2015; Li et al., 2013), with implications for relapse risk (Andó et al., 2012; Paulus et al., 2005) and long-term recovery (Wang et al., 2013). Abstinence from a particular drug of choice was indicated as currently using, 1 month, 3 months, 6 months, or more than 1 year of abstinence, and participants were further divided into current (≤ 1 month abstinence) vs. recovering ( ≥3 months abstinence) users. This division aligns with the DMS 5 criteria for early remission in substance use disorders (American Psychiatry Association, 2025) and with neural and metabolic alterations associated with abstinence (Bell et al., 2011; Beveridge et al., 2009; Lu et al., 2014; Nicolas et al., 2017; Sourty et al., 2024; Wang et al., 2012). Ages and genders for each recruited substance, for current and recovering uses, are shown in Table 1. Demographic variables were included in all statistical models.

**Table 1:** Demographic information and number of individuals in each drug category

Main Drug Category	Abstinence	Age	Females	Total
Psychostimulants	Current	38 ± 10	30	38
	Recovering	42 ± 14	30	49
Opioids	Current	34 ± 11	16	25
	Recovering	40 ± 11	33	48
Sedatives	Current	35 ± 11	12	23
	Recovering	40 ± 12	28	38
Alcohol	Current	35 ± 13	27	62
	Recovering	43 ± 12	32	50

Following the abstinence query, which was presented immediately upon task completion, a composite severity questionnaire was administered, consisting of, in order, the duration of the last period of use (1 month, 3 months, 6 months, 12 months, or longer than 12 months), substance risk scores from the online-adapted *NIDA Quick Screen Version 1* for each substance, and from an online-adapted version of the DSM-5 Substance Use Disorder criteria. An SVD was performed on the scores from each severity component to obtain a single severity score. These scores were included in all statistical models, together with the demographic variables listed in Table 1.

### Task & Procedure

The task was as illustrated in Fig. 1, with the commitment, at the start of each of several gambling rounds, to one of two “rooms” in which to gamble for the duration of that round serving as the primary measure. The room-choice screens were as illustrated in Fig. 1a, with pie charts representing the color outcome distributions of two alternative gambling options inside each room and with explicit labels indicating free vs. forced choice, denoted in the study as “self-play” and “auto-play,” respectively. Participants were instructed that they would be allowed to choose freely between options in “self-play” rooms but that the computer would alternate between options across trials in “auto-play” rooms. They were further told that the monetary values of color outcomes would change unpredictably across rounds (mimicking the fluctuating utilities of natural rewards) and only be revealed once a room had been selected. Their objective, thus, was to select, in each round, the gambling room that would allow them to maximize monetary payoffs in that round, given only information about the affordance of instrumental control, free choice, and outcome divergence in each room.

Once a room had been selected, the monetary value of each color in the round was revealed, and four discrete gambling trials were performed in the room, with choice, selection, and feedback screens as illustrated in Fig. 1b. Computer-selected gambling options (i.e., forced choices) were indicated by a dashed square outline, and participants had to press the corresponding key in order to progress through the trial. The divergence of color outcome distributions associated with the gambling options inside a room was quantified as their information theoretic distance (see Eq. 1), with four distances (0.00, 0.04, 0.15, and 0.2) yielding six unique *differences* in outcome divergence across gambling rooms. Each pair of outcome divergences was combined with each self- vs. auto-play combination for a total of 24 unique room-choice scenarios, and these were repeated across two color schemes for 48 total room-choice trials and 192 total gambling trials.

Recall that, in the absence of free choice, high outcome divergence allows *prediction but not control* of outcomes. Comparisons of different levels of outcome divergence across rooms that were both self-play (or both auto-play), and those with the same outcome divergence but differing on self- vs auto-play, allowed for assessment of independent preferences for outcome divergence and free choice. Also note that if instrumental control was assessed with respect to reward rather than sensory features, it would constantly fluctuate according to homeostatic changes in subjective utilities, mimicked here by unpredictable changes in the monetary payoffs associated with color outcomes. To ensure that monetary color outcomes, which ranged from − $2 to $3 in $1 increments, could not account for a controllability preference, they were precoded using a semirandomly assignments to colors across rounds with the constraint that low divergence rooms must have equal or higher expected payoffs than high divergence rooms, and free-choice rooms must have equal or lower expected payoffs than forced-choice rooms. Finally, to maintain incentive compatibility across repeated exposure to monetary outcomes (Botvinik-Nezer et al., 2019), participants were instructed that they would get to keep the monetary earnings, up to $15, from only two gambling rounds, drawn randomly at the end of the study.

### Computational variables

An environment’s controllability has been formalized as the Jensen-Shannon divergence (Lin, 1991) of the outcome probability distributions associated with available actions (Liljeholm et al., 2013). For the case illustrated in Fig. 1a, let *P*_*1*_ and *P*_*2*_ be the respective color probability distributions of the two options available in a given room, let *O* be the set of possible color outcomes, and *P(o)* the probability of a particular color outcome. The divergence between options in a room with respect to their color outcome distribution, henceforth outcome divergence (OD), is:

1 $$OD=\frac{1}{2}{\sum }_{o\in O}ln\left(\frac{{P}_{1}\left(o\right)}{{P}_{*}\left(o\right)}\right){P}_{1}(o)+\frac{1}{2}{\sum }_{o\in O}ln\left(\frac{{P}_{2}\left(o\right)}{{P}_{*}\left(o\right)}\right){P}_{2}\left(o\right),$$

where *ln* is the natural logarithm, yielding nats, and ${P}_{*}\left(o\right)=\frac{1}{2}({P}_{1}+{P}_{2})$.

Recall that, while an information theoretic divergence can be computed over distributions associated with any number and type of random variable(s), it only reflects an environment’s *controllability* when computed over sensory outcome distributions associated with freely chosen actions (Liljeholm, 2022).

We specify three competing models of expected room utility that differ with respect to their treatment of free choice and controllability. In all three models, the value of a particular gambling room (i.e., a particular pair of pie charts) was incrementally updated across gambling rounds based on monetary payoffs, such that

2 $${E\$}_{ij+1}\leftarrow \alpha *{\Sigma \$}_{ij}+\left(1-\alpha \right)*{E\$}_{ij}$$

where ${E\$}_{ij+1}$ is the expected monetary pay-off in room *i* at the start of round *j* + *1* (i.e., at the start of the next round played in room *i*)*,* ${\Sigma \$}_{ij}$ is the sum of monetary outcomes earned in room *i* at the end of round *j* (i.e., the end of the current round)*,* ${E\$}_{ij}$ is the expected monetary payoff in room *i* on round *j* (recursively estimated based on the payoff, ${\Sigma \$}_{ij-1}$, and expectation, ${E\$}_{ij-1}$, in the previous round, *j-1*)*,* and α is a free learning rate parameter, fit to each individual participant.

In a *Baseline* model, E$ fully accounted for the expected value of a room, characterizing a model-free agent that maximizes rewards solely based on experienced monetary payoffs. This model scales with the coded monetary payoffs for each condition, which were constrained to dissociate the control preference from monetary reward. In each of two alternative models, an additional term was added to reflect intrinsic motivation. In the *Free Choice* model, instances of free vs. forced choice (F) were coded as 1 and 0 respectively, with free parameter γ estimating the subjective utility of free choice. In the *Control* model, F was multiplied with the degree of outcome divergence (D) afforded by the room:

3 $${V}_{Room}=E\$+\gamma FD$$

Model-derived room values were transformed into room choice probabilities using a softmax rule with a noise parameter, τ.

4 $$p\left({room}_{i}\right)=\frac{{e}^{V{room}_{i}*\tau }}{{\sum }_{j=1}^{n}{e}^{V{room}_{j}*\tau }}$$

Free model parameters were fit to behavioral data by minimizing the negative log likelihood and model comparisons were performed on the corrected Akaike Information Criterion (AIC), which penalizes complexity. Parameter recovery was assessed by simulating 1,000 gamblers, each with a set of parameter values drawn randomly from the range used in model fitting, and each receiving a sequence of room options identical to that of a randomly drawn participant, with the sequence repeated 10 times (yielding 480 rounds) to ensure sufficient training. The data-generating model was then used to estimate parameters from the simulated data, and Pearson’s correlation coefficients were used to quantify recovery. All modeling and statistical analyses were performed using MATLAB (mathworks.com).

## Results

**Fig. 2:**
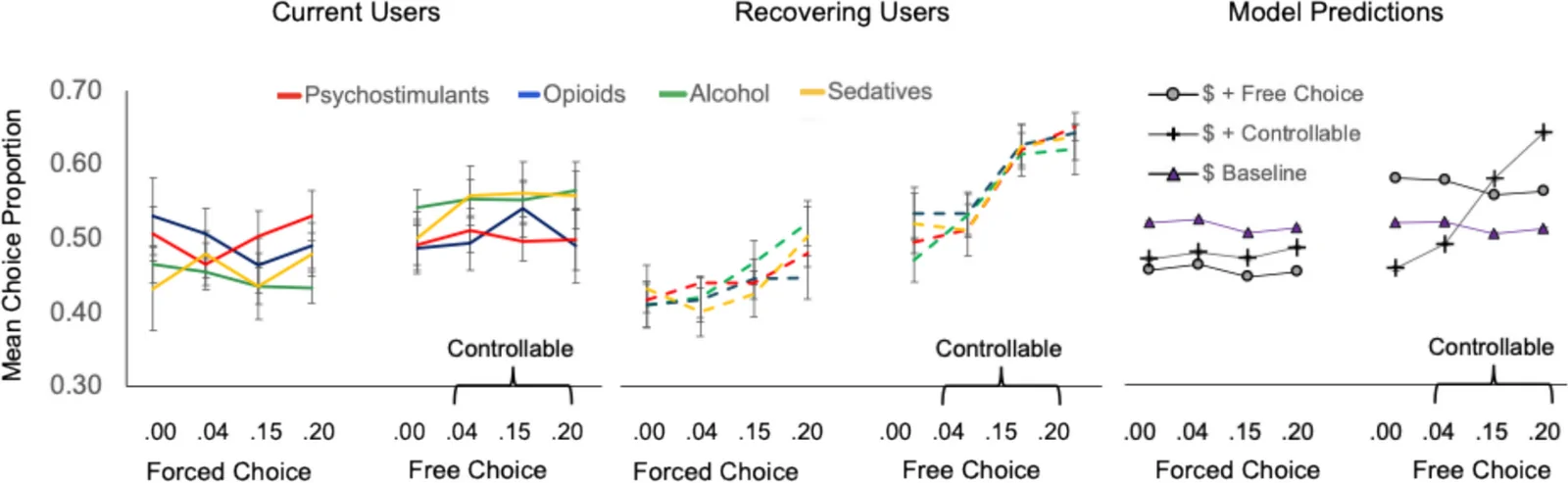
Choice proportions and model predictions. *Note.* Mean choice preferences across gambling rooms as a function of Free Choice, Outcome Divergence, and Controllability, in Current (left) and Recovering (middle) Users for each substance, together with predictions from model simulations (right) with a monetary Baseline model (triangle), a Free Choice model (circle), and a Control (+) model. Error bars = SEM

Of particular interest was how Abstinence from Psychostimulant, Opioid, Alcohol, and Sedative use respectively related to preferences for instrumental control. Mean choice preferences for gambling environments as a function of Free Choice, Outcome Divergence, and Controllability, in Current (use in the last 1 month) and Recovering (no use for more than 3 months) users, are plotted in Fig. 2, for each substance category. Strikingly, across substances, Current users do not display the characteristic preference for Controllability clearly seen in Recovering users, as well as in previous studies using healthy volunteers (Liljeholm, 2022).

A control-preference score was computed for each participant as the ratio of choice proportions for rooms with control (free choice & non-zero divergence) over that for rooms without control and was entered as the outcome variable in a Linear Mixed Effects (LME) model. The LME allowed us to assess differences across substances (i.e., psychostimulants, opioids, etc.) despite the data being unbalanced by polysubstance use. The model further specified abstinence (current vs. recovering), severity, substance, polysubstance use, gender, and age as fixed effects, and participant as random effect. We found a significant abstinence-by-substance interaction (t_325_ = 3.25, *p* <.01 [95% CI 0.03, 0.13]), as well as significant effects of Abstinence (t_325_ =  − 4.63, *p* <.001 [95% CI − 0.42, − 0.17]) and Substance (t_325_ =  − 2.52, *p* <.05 [95% CI − 0.09, − 0.01]). No other effects were significant (see Table 2 for all results). Critically, the same fixed and random effects modeling the ratio of the mean amount of dollar outcomes generated by participants in controllable rooms and the expectation given optimal selection revealed only a significant effect of polysubstance use (t_321_ =  − 2.95, *p* <.01 [95% CI − 0.10, − 0.02]), but no other significant effects (Table 2).

**Table 2:** Statistical output from linear mixed-effects models of the control preference and of monetary reward maximization, with current vs. recovering users, use severity, polysubstance use, gender, age, & substance used as fixed effects and participant as random effect

Outcome variable	Predictors	*df*	*t*	*p*
*Control preference*	Current vs. recovering (abstinence)	325	− 4.63	< *0.001*
	Severity	325	− 0.40	0.69
	Substance	325	− 2.52	0.01
	Polysubstance use	325	− 0.10	0.92
	Gender	325	0.73	0.47
	Age	325	− 1.23	0.22
	Abstinence x substance	325	3.25	< *0.01*
*Reward Maximization*	Current vs. recovering (abstinence)	321	− 0.46	0.65
	Severity	321	− 1.07	0.28
	Substance	321	− 0.64	0.52
	Polysubstance use	321	− 2.95	< *0.01*
	Gender	321	− 0.27	0.79
	Age	321	0.12	0.90
	Abstinence x substance	321	− 0.05	0.96

Although the LME identified Abstinence as the only variable that significantly interacted with the type of substance to predict a controllability preference, it weighs variables according to their unique contributions, so it is possible that shared variance occluded similar effects for other predictors in the model. To assess this possibility, we ran five LMEMs, each with substance as a fixed effect and participant as a random effect, and each assessing only one additional predictor of the control preference: abstinence, severity, poly-use, gender, or age. As in the full model, abstinence (t_329_ =  − 4.38, *p* < 0.001) and the abstinence × substance interaction (t_329_ = 2.87, *p* < 0.01) were significant predictors in these models. Moreover, the poly-use variable significantly predicted the control preference when not competing (t_329_ =  − 1.96, *p* = 0.05), as did age (t_329_ =  − 2.55, *p* = 0.01), while the substance variable was only marginally significant in the model that included abstinence (t_329_ =  − 1.73, *p* = 0.08) and did not reach significance in any model that lacked the abstinence predictor (all *p* > *.*12; see supplemental Table 3 for full results).

**Table 3:** Mean best fitting parameters by utility model (baseline, choice, & control), abstinence (current vs. recovering), and substance

		Current			Recovering		
Substance	Model	α	τ	γ	α	τ	γ
Psychostimulants	Baseline	0.16	0.46	-	0.23	0.46	-
	Choice	0.12	0.65	0.44	0.12	0.73	0.63
	Control	0.19	0.42	0.19	0.27	0.43	0.35
Opioids	Baseline	0.11	0.56	-	0.20	0.47	-
	Choice	0.10	0.67	0.44	0.11	0.77	0.68
	Control	0.17	0.47	0.18	0.22	0.42	0.32
Sedatives	Baseline	0.22	0.37	-	0.11	0.59	-
	Choice	0.17	0.59	0.55	0.04	0.89	0.63
	Control	0.29	0.28	0.22	0.14	0.55	0.32
Alcohol	Baseline	0.11	0.55	-	0.21	0.53	-
	Choice	0.09	0.82	0.55	0.11	0.82	0.61
	Control	0.14	0.50	0.22	0.25	0.52	0.32

To further probe the abstinence-by-substance interaction for the control preference, and to model the full factorial design, separate mixed analyses of variance (ANOVA) were performed for each substance, with outcome divergence and free choice as within-subjects variables, current vs. recovering users as a between-subject variable, and with use severity, poly-use, gender, and age as covariates. These analyses yielded a significant abstinence-by-outcome divergence-by-free choice interaction (*F*_*3,243*_ = 4.67, *p* < 0.01), as well as an abstinence-by-free choice interaction (*F*_*1,81*_ = 5.00, *p* = 0*.*03), for psychostimulant use only. For opioid use, there was an abstinence-by-free choice (*F*_*1,67*_ = 3.86, *p* = 0.05) interaction, and for alcohol use an abstinence-by-outcome divergence interaction (*F*_*3,318*_ = 3.03, *p* = 0.03). No significant effects involving abstinence emerged for sedative users (all *p* > 0.34; see Supplemental Table 1 for all effects).

For a more fine-grained analysis, we specified three expected utility models that generated choice preferences across gambling environments. Predictions from model simulations, averaged across the parameter space, are plotted in Fig. 2. Briefly, in a Baseline model, the expected utility of a particular gambling room depended solely on its previous monetary payoffs. In Choice and Control models, room utilities depended on the intrinsic subjective utility of free choice and instrumental control, respectively, in addition to monetary payoffs. Mean best-fitting parameter estimates, across models, substances, and abstinence levels, are listed in Table 3. Except for marginally significant effects for the monetary learning rate (α) in psychostimulant use (*p* =.08) and the decision noise (τ) in alcohol use (*p* =.09), pairwise comparisons revealed no significant differences between parameter estimates based on abstinence levels (all *p* >.14). Parameter recovery was excellent across models, for the monetary learning rate (*r* > 0.99 and *p* < 0.001), decision noise (*r* > 0.99 and *p* < 0.001), and the subjective utility of control (or free choice, depending on model; *r* > 0.91 and *p* < 0.001).

AIC scores from model fitting are listed in Table 4. We specified three LMEMs, each with the difference between two model AIC scores (i.e., 1. control vs. baseline, 2. control vs. choice, & 3. choice vs. baseline), as the outcome variable, and with current vs. recovering user, use severity, main use substance, poly-use, gender, and age as fixed effects, and participant as random effect. For the control vs. baseline model, we again found significant effects of abstinence (t_325_ =  − 3.51, *p* < 0.001) and substance (t_325_ =  − 2.05, *p* <.05) as well as a significant abstinence-by-substance interaction (t_325_ = 2.25, *p* = 0.03). For the control vs. choice model, there was again a significant effect of abstinence (t_325_ =  − 2.53, *p* < 0.05), but the abstinence-by-substance interaction did not reach significance (*p* = 0.16), nor did any other effects. Finally, for the choice vs. baseline model, only gender emerged as a significant predictor (t_325_ = 3.17, *p* < 0.005). All results from these comparisons are listed in Table 5.

**Table 4:** Mean AIC scores by utility model (baseline, choice, & control), abstinence, and substance

	Current			Recovering		
	Baseline	Choice	Control	Baseline	Choice	Control
Psychostimulants	68.02	67.64	67.86	67.79	66.80	63.79
Opioids	67.32	66.95	66.40	68.29	66.22	65.26
Sedatives	67.98	66.73	66.67	67.70	65.68	64.59
Alcohol	67.96	65.84	66.63	67.21	65.61	63.82

**Table 5:** Statistical output from linear mixed effects models of the control preference and of monetary reward maximization, with current vs. recovering users, use severity, polysubstance use, gender, age, & substance used as fixed effects and participant as random effect

Outcome variable	Predictors	*df*	*t*	*p*
*Control vs. baseline*	Current vs. recovering (abstinence)	325	− 3.53	< 0.001
	Severity	325	0.79	0.43
	Substance	325	− 2.06	0.04
	Polysubstance use	325	− 0.27	0.78
	Gender	325	1.36	0.18
	Age	325	− 0.82	0.41
	Abstinence x substance	325	2.31	0.02
*Control vs. choice*	Current vs. recovering (abstinence)	321	− 2.48	0.01
	Severity	321	1.63	0.10
	Substance	321	− 0.31	0.76
	Polysubstance use	321	− 1.04	0.30
	Gender	321	− 1.70	0.09
	Age	321	− 0.13	0.90
	Abstinence x substance	321	1.37	0.17
*Choice vs. baseline*	Current vs. recovering (abstinence)	325	− 1.70	0.09
	Severity	325	− 0.80	0.42
	Substance	325	− 1.57	0.12
	Polysubstance use	325	1.07	0.28
	Gender	325	3.25	< 0.01
	Age	325	− 0.56	0.58
	Abstinence x substance	325	0.85	0.39

Planned comparisons performing the same regression separately on each substance again revealed significant effects only of abstinence from psychostimulant use, which predicted a significantly better fit by the control than the baseline model (t_81_ =  − 3.07, *p* <.01 [95% CI − 0.75, − 0.16]) as well as the control than the choice model (t_81_ =  − 2.94, *p* <.01 [95% − 0.57, − 0.11]). No other effects of abstinence were found for any substances, but gender and poly-use emerged as significant predictors for psychostimulant (t_81_ = 2.22, *p* =.03 [95% CI 0.19, 3.50]) and alcohol (t_106_ = 2.2, *p* = 0.03, [95% CI 0.29, 5.62]) use respectively when comparing baseline and choice models (see Supplemental Table 2).

## Discussion

We combined a hierarchical gambling task with cross-sectional substance use surveys and computational cognitive modeling to assess the preference for controllable environments in adult human participants with self-reported durations of abstinence from psychostimulant, opioid, alcohol, and sedative use. For psychostimulant use, abstinence strongly modulated the preference for controllable environments. In contrast, for alcohol, the recency of use was more predictive of a preference for environments with divergent outcome distributions across available options, regardless of whether those outcomes were controllable. Critically, these associations between substance use and control preferences were dissociable from participants’ ability to maximize monetary payoffs in controllable environments.

Since the subjective utilities of sensory states are constantly fluctuating, reward maximization depends critically on the ability to flexibly produce or prevent states according to their current utilities. Consistent with this notion, neurotypical human decision-makers prefer, all else being equal, to act in environments with greater controllability (Liljeholm, 2022; Liljeholm et al., 2018; Mistry & Liljeholm, 2016). This preference, which was observed here in recovering but not current psychostimulant users, has been shown to scale with activity in the ventromedial prefrontal cortex (vmPFC; Norton & Liljeholm, 2020), an area heavily implicated in domain general decision-value computations (Grabenhorst & Rolls, 2011; Krawczyk, 2002; O’Doherty, 2011) and to decrease with *positive schizotypy* (Liljeholm, 2022; Liljeholm et al., 2018)*:* a subclinical analogue of positive schizophrenia (Mason & Claridge, 2006). Together, these findings hint at a shared dopaminergic (DA) basis for the control preference: the vmPFC is a major output region for dopamine-producing neurons in the ventral tegmental area (Han et al., 2017), and DA aberrations are well documented in positive schizotypy and schizophrenia (Abi-Dargham & Grace, 2011; Dawe et al., 2013; Guillin et al., 2007; Mohr & Ettinger, 2014; Woodward et al., 2011).

A DA interpretation of the control preference is consistent with our findings that abstinence effects were most pronounced with psychostimulant use, specifically amphetamine and cocaine. Unlike opioids and alcohol, which increase DA by increasing the firing of dopamine neurons, amphetamine and cocaine act directly on the dopamine terminal, respectively mobilizing the vesicular release from and blocking the transport back into the presynaptic cell (Di Chiara & Imperato, 1988). Accordingly, DA concentrations in the nucleus accumbens of freely moving rats following systemic injections of illicit drugs were found to be elevated much more by Amphetamine and (to lesser extent) cocaine than by opioids, which in turn were more robust than those produced by alcohol (Di Chiara & Imperato, 1988). Conversely, all substances assessed here, including sedatives, are well known to produce significant DA elevations (Boileau et al., 2003; Di Chiara & Imperato, 1986, 1988; Spanagel et al., 1990), so no strong claims about the selectivity of psychostimulants are warranted based on the DA hypothesis of control, nor does our data support such categorical divisions.

The interpretation of our results is constrained by several study limitations. First, given our use of cross-sectional measures, although we propose that abstinence may facilitate a recovery of the subjective value of control, the reverse causal direction—that a higher inherent preference for control promotes successful abstinence—remains a distinct possibility. Second, there is substantial overlap across substances used in our sample, and indeed in the population, which make independent effects exceedingly difficult to discern. Third, our abstinence measure, though aligned with identified remission criteria, had limited resolution. Finally, we did not obtain independent clinical verification of reported substance use. Future studies will need to address these factors to further characterize the role of instrumental control, and agency more broadly, in substance use disorder. Nevertheless, the current work has identified several core distinctions with respect to the influence of substance-use on the preference for instrumental control, including that between different illicit substances, and corollary neuromodulatory systems, between dissociable subcomponents of instrumental control, such as free choice and outcome divergence, and between the utility of control and basic monetary reward maximization.

## Supplementary Information

Below is the link to the electronic supplementary material.

Supplementary file1 (DOCX 28 KB)

## Data Availability

All data and study materials are publicly available (https://osf.io/yj7uk/?view_only=af1bc26fe6454bd6b85b2de05fb7ffde).

## Code availability

All code is publicly available (https://osf.io/yj7uk/?view_only=af1bc26fe6454bd6b85b2de05fb7ffde).
