# Supplementary material for: Substance use and the value of control
Source: Cogn Affect Behav Neurosci. 2026 May 6;26(5):2311–20. doi: 10.3758/s13415-026-01451-z (PMC13615113; doi:10.3758/s13415-026-01451-z)
Supplement: Supplementary file 1 [file 13415_2026_1451_MOESM1_ESM.docx]

**Supplemental Table 1***.* Statistical output from Mixed ANOVAs, one per substance, assessing the influence of Abstinence, outcome Divergence, and free Choice on gambling room preferences, with use Severity, Polysubstance (Poly) Use, Age and Gender as covariates.

| Main Drug Category | Predictors | *df* | *F* | *p* |
| --- | --- | --- | --- | --- |
| Psychostimulants | Choice | 1,81 | 1.40 | 0.24 |
|  | Choice x Abstinence | 1,81 | 4.99 | *0.03* |
|  | Choice x Severity | 1,81 | 0.29 | 0.59 |
|  | Choice x Poly Use | 1,81 | 0.95 | 0.33 |
|  | Choice x Age | 1,81 | 1.36 | 0.25 |
|  | Choice x Gender | 1,81 | 1.76 | 0.19 |
|  | Divergence | 3,243 | 2.57 | 0.05 |
|  | Divergence x Abstinence | 3,243 | 0.78 | 0.51 |
|  | Divergence x Severity | 3,243 | 2.09 | 0.10 |
|  | Divergence x Poly | 3,243 | 0.45 | 0.71 |
|  | Divergence x Age | 3,243 | 1.17 | 0.32 |
|  | Divergence x Gender | 3,243 | 0.95 | 0.42 |
|  | Choice x Divergence | 3,243 | 0.96 | 0.41 |
|  | Choice x Divergence x Abstinence | 3,243 | 4.67 | *<0.01* |
|  | Choice x Divergence x Severity | 3,243 | 0.92 | 0.43 |
|  | Choice x Divergence x Poly | 3,243 | 0.03 | 0.99 |
|  | Choice x Divergence x Age | 3,243 | 2.24 | 0.08 |
|  | Choice x Divergence x Gender | 3,243 | 1.09 | 0.35 |
| Opioids | Choice | 1,67 | 0.30 | 0.59 |
|  | Choice x Abstinence | 1,67 | 3.86 | *0.05* |
|  | Choice x Severity | 1,67 | 0.17 | 0.68 |
|  | Choice x Poly | 1,67 | 0.24 | 0.62 |
|  | Choice x Age | 1,67 | 1.47 | 0.23 |
|  | Choice x Gender | 1,67 | 0.28 | 0.60 |
|  | Divergence | 3,201 | 0.66 | 0.57 |
|  | Divergence x Abstinence | 3,201 | 0.90 | 0.44 |
|  | Divergence x Severity | 3,201 | 0.76 | 0.52 |
|  | Divergence x Poly Use | 3,201 | 0.76 | 0.52 |
|  | Divergence x Age | 3,201 | 0.35 | 0.79 |
|  | Divergence x Gender | 3,201 | 1.99 | 0.12 |
|  | Choice x Divergence | 3,201 | 2.85 | *0.04* |
|  | Choice x Divergence x Abstinence | 3,201 | 0.32 | 0.81 |
|  | Choice x Divergence x Severity | 3,201 | 0.20 | 0.89 |
|  | Choice x Divergence x Poly | 3,201 | 0.89 | 0.45 |
|  | Choice x Divergence x Age | 3,201 | 3.13 | *0.03* |
|  | Choice x Divergence x Gender | 3,201 | 2.26 | 0.08 |
| Sedatives | Choice | 1,55 | 2.07 | 0.16 |
|  | Choice x Abstinence | 1,55 | 0.92 | 0.34 |
|  | Choice x Severity | 1,55 | 0.20 | 0.66 |
|  | Choice x Poly Use | 1,55 | 2.08 | 0.16 |
|  | Choice x Age | 1,55 | 0.45 | 0.51 |
|  | Choice x Gender | 1,55 | 0.23 | 0.64 |
|  | Divergence | 3,165 | 1.59 | 0.19 |
|  | Divergence x Abstinence | 3,165 | 1.13 | 0.34 |
|  | Divergence x Severity | 3,165 | 0.31 | 0.82 |
|  | Divergence x Poly Use | 3,165 | 1.61 | 0.19 |
|  | Divergence x Age | 3,165 | 1.06 | 0.37 |
|  | Divergence x Gender | 3,165 | 1.68 | 0.17 |
|  | Choice x Divergence | 3,165 | 0.21 | 0.89 |
|  | Choice x Divergence x Abstinence | 3,165 | 0.28 | 0.84 |
|  | Choice x Divergence x Severity | 3,165 | 0.49 | 0.69 |
|  | Choice x Divergence x Poly Use | 3,165 | 0.21 | 0.89 |
|  | Choice x Divergence x Age | 3,165 | 0.85 | 0.47 |
|  | Choice x Divergence x Gender | 3,165 | 0.09 | 0.96 |
| Alcohol | Choice | 1,106 | 3.66 | 0.06 |
|  | Choice x Abstinence | 1,106 | 0.09 | 0.76 |
|  | Choice x Severity | 1,106 | 0.14 | 0.71 |
|  | Choice x Poly | 1,106 | 0.12 | 0.73 |
|  | Choice x Age | 1,106 | 0.26 | 0.61 |
|  | Choice x Gender | 1,106 | 1.81 | 0.18 |
|  | Divergence | 3,318 | 0.65 | 0.58 |
|  | Divergence x Abstinence | 3,318 | 3.03 | *0.03* |
|  | Divergence x Severity | 3,318 | 0.20 | 0.90 |
|  | Divergence x Poly Use | 3,318 | 0.17 | 0.92 |
|  | Divergence x Age | 3,318 | 0.26 | 0.85 |
|  | Divergence x Gender | 3,318 | 0.66 | 0.58 |
|  | Choice x Divergence | 3,318 | 2.20 | 0.09 |
|  | Choice x Divergence x Abstinence | 3,318 | 0.43 | 0.73 |
|  | Choice x Divergence x Severity | 3,318 | 2.12 | 0.10 |
|  | Choice x Divergence x Poly Use | 3,318 | 1.56 | 0.20 |
|  | Choice x Divergence x Age | 3,318 | 2.75 | *0.04* |
|  | Choice x Divergence x Gender | 3,318 | 0.97 | 0.41 |

**Supplemental Table 2***.* Results from linear regressions, one per substance, assessing the relationship between Abstinence and computational model fits for Baseline (B), Choice (Cho), & Control (Ctrl), with use Severity, Poly Use, Age, & Gender as covariates.

|  |  | Ctrl– Cho | | Cho - B | | Ctrl - B | |
| --- | --- | --- | --- | --- | --- | --- | --- |
| Main Drug Category | Variables | t | *p* | t | *p* | t | *p* |
| Psychostimulants | Length of Abstinence | -2.94 | *<0.01* | -1.32 | 0.19 | -3.07 | *<0.01* |
|  | Severity | 0.02 | 0.98 | -0.93 | 0.35 | -0.53 | 0.60 |
|  | Poly Use | -0.08 | 0.93 | 0.54 | 0.59 | 0.25 | 0.80 |
|  | Age | -0.10 | 0.92 | -0.85 | 0.40 | -0.58 | 0.56 |
|  | Gender | 0.77 | 0.45 | 2.22 | *0.03* | 1.90 | 0.06 |
| Opioids | Length of Abstinence | -0.57 | 0.57 | -0.84 | 0.40 | -1.09 | 0.28 |
|  | Severity | 0.07 | 0.94 | -0.15 | 0.88 | -0.04 | 0.97 |
|  | Poly Use | 1.76 | 0.08 | -0.27 | 0.78 | 1.32 | 0.19 |
|  | Age | -0.01 | 0.99 | -0.09 | 0.93 | -0.08 | 0.94 |
|  | Gender | 1.20 | 0.24 | 0.44 | 0.66 | 1.34 | 0.18 |
| Sedatives | Length of Abstinence | -0.98 | 0.33 | -0.34 | 0.74 | -1.17 | 0.25 |
|  | Severity | -0.38 | 0.71 | -0.97 | 0.34 | -1.07 | 0.29 |
|  | Poly Use | 0.47 | 0.64 | -1.32 | 0.19 | -0.54 | 0.59 |
|  | Age | -0.77 | 0.44 | 0.59 | 0.56 | -0.29 | 0.78 |
|  | Gender | -0.01 | 0.99 | 0.34 | 0.74 | 0.24 | 0.81 |
| Alcohol | Length of Abstinence | -1.38 | 0.17 | -0.58 | 0.56 | -1.64 | 0.10 |
|  | Severity | 0.77 | 0.44 | -0.24 | 0.81 | 0.45 | 0.65 |
|  | Poly Use | -1.48 | 0.14 | 2.20 | *0.03* | 0.54 | 0.59 |
|  | Age | -0.74 | 0.46 | 1.31 | 0.19 | 0.44 | 0.66 |
|  | Gender | 0.13 | 0.90 | 1.51 | 0.13 | 1.34 | 0.18 |

**Supplemental Table 3***.* Results of five linear mixed effects models predicting Control Preference. Each model included Substance as a fixed effect and participant as a random effect, with separate models estimated for each predictor: Abstinence Status, Use Severity, Polysubstance Use, Gender, and Age.

| Model | Predictors | *df* | *t* | *p* |
| --- | --- | --- | --- | --- |
| Abstinence | Abstinence | 329 | -4.38 | *<0.001* |
|  | Substance | 329 | -1.73 | 0.08 |
|  | Abstinence x Substance | 329 | 2.87 | *<0.01* |
| Severity | Severity | 329 | 1.07 | 0.28 |
|  | Substance | 329 | -1.42 | 0.16 |
|  | Severity x Substance | 329 | -1.58 | 0.12 |
| Polysubstance Use | Polysubstance Use | 329 | -1.96 | *0.05* |
|  | Substance | 329 | -1.36 | 0.17 |
|  | Polysubstance Use x Substance | 329 | 1.42 | 0.16 |
| Gender | Gender | 329 | 0.19 | 0.85 |
|  | Substance | 329 | -0.34 | 0.74 |
|  | Gender x Substance | 329 | 0.49 | 0.63 |
| Age | Age | 329 | -2.55 | *0.01* |
|  | Substance | 329 | -0.64 | 0.52 |
|  | Age x Substance | 329 | 0.75 | 0.45 |
